# Modified medial minimally invasive double-plating osteosynthesis techniques for the treatment of distal third diaphyseal fracture of humerus

**DOI:** 10.1038/s41598-023-49111-3

**Published:** 2023-12-07

**Authors:** Youyou Ye, Yanbin Lin, Chunling Wu, Yunzhe Zhu

**Affiliations:** Present Address: Department of Traumatic Orthopaedics, Fuzhou Second Hospital, The Third Clinical Medical College, Fujian Medical University, 47th Shangteng Road of Cangshan District, Fuzhou, 350007 Fujian China

**Keywords:** Diseases, Trauma

## Abstract

The optimal surgical approach and placement of plates for the treatment of distal third diaphyseal fracture of the humerus are the subjects of debate. The aim of this retrospective study was to evaluate the clinical and radiographic outcomes of modified medial minimally invasive plate osteosynthesis (MIPO) techniques using a double technique for the treatment of distal third diaphyseal fracture of the humerus. A total of 30 patients with a distal third diaphyseal fracture of the humerus were selected from our hospital. Patients were seen between January 2017 and October 2022. They were treated with a modified medial approach combined with MIPO using a double plate technique. Patient demographics, operation time, bleeding volume, union time, complications, the mean fracture length (FL) and distal cortical length (DCL), and the number of screws in the distal fragment were analyzed. The function of the shoulder and elbow was evaluated using Neer’s assessment of the shoulder and Mayo’s assessment of the elbow. The FL was 56.1 ± 7.2 mm and the DCL was 38.3 ± 5.3 mm. The mean operative time was 84.8 ± 13.4 min (range 60–110 min). The mean blood loss during surgical treatment was 46.5 ± 10.2 ml (range 30–60 ml). Bone healing was observed in all patients from 10 to 16 weeks (average 12.1 ± 1.7) postoperatively, and one case with poor surgical wound healing was recorded. All the patients had good function of both the shoulder and elbow. The maximum flexibility of the elbow ranged from 130° to 145° (average 138.1 ± 4.8°), with a maximum flexibility straightness ranging from 0° to 5° (average 2.2 ± 1.3°). The Mayo elbow joint function score was 80–100 (average 91.4 ± 5.0). The Neer shoulder joint function score ranged from 85 to 100 (average 92.5 ± 3.9). The modified medial approach was beneficial it did not cause any iatrogenic radial nerve or ulnar nerve injuries. The anterior and the medial side plates are fixed perpendicular to the distal humerus and provide excellent stability at the same time producing better shoulder and elbow joint function.

## Introduction

A distal third diaphyseal fracture of the humerus is often caused by indirect violence and has an oblique or spiral shape. Due to high rates of nonunion and radial nerve injury, the treatment of this kind of fracture is difficult^1^. Surgical treatment, including external fixation or an intramedullary nail and plate placement, can be selected. It is generally considered that open reduction and internal fixation with a plate is the most reliable treatment method^2^. Single plate fixation is the choice among most surgeons^3,4^. However, it was reported^5^ that the single plate fixation humeral shaft nonunion and internal fixation failure rate can reach 15%. Therefore, to reduce the risk of complications of such fractures, the distal fracture needs to be fixed by at least three or four screws to achieve good stability, however, it is obvious that only a double plate can accommodate such a fracture. Some physicians believe that this kind of fracture should be treated with double plates^6,7^. Approaches for treating distal third diaphyseal fractures of the humerus include anterior^8^, anterolateral^9^, and posterior^10^ approaches, each of which has advantages and disadvantages. An anteromedial modified medial minimally invasive plate osteosynthesis (MIPO) approach that can be performed through the internervous plane beneath the brachialis muscle without exposing any nerves or causing any muscle splitting with a 12-hole plate was reported, which permitted less invasive surgical dissection, allowed the use of longer distal screws and achieved better cosmesis^11^. However, this approach is limited in its ability to expose the anterior of the distal humerus, especially when plate fixation is required. Therefore, a modified medial approach was proposed to provide a greater range of exposure in our study. The purpose of this study was to describe this modified medial approach combined with MIPO using a double-plate technique for the treatment of a distal third diaphyseal fracture of the humerus and to assess its clinical outcome.

## Materials and methods

The Human Experimentation and Ethics Committee of our hospital’s institutional review board gave its approval for the study design and data collection (2023071). This study was conducted in accordance with the ethical standards of the 1964 Declaration of Helsinki and its amendments. Informed consent was obtained from all individual participants included in the study**.**

### Patient selection and radiographic measurements

**Figure 1:**
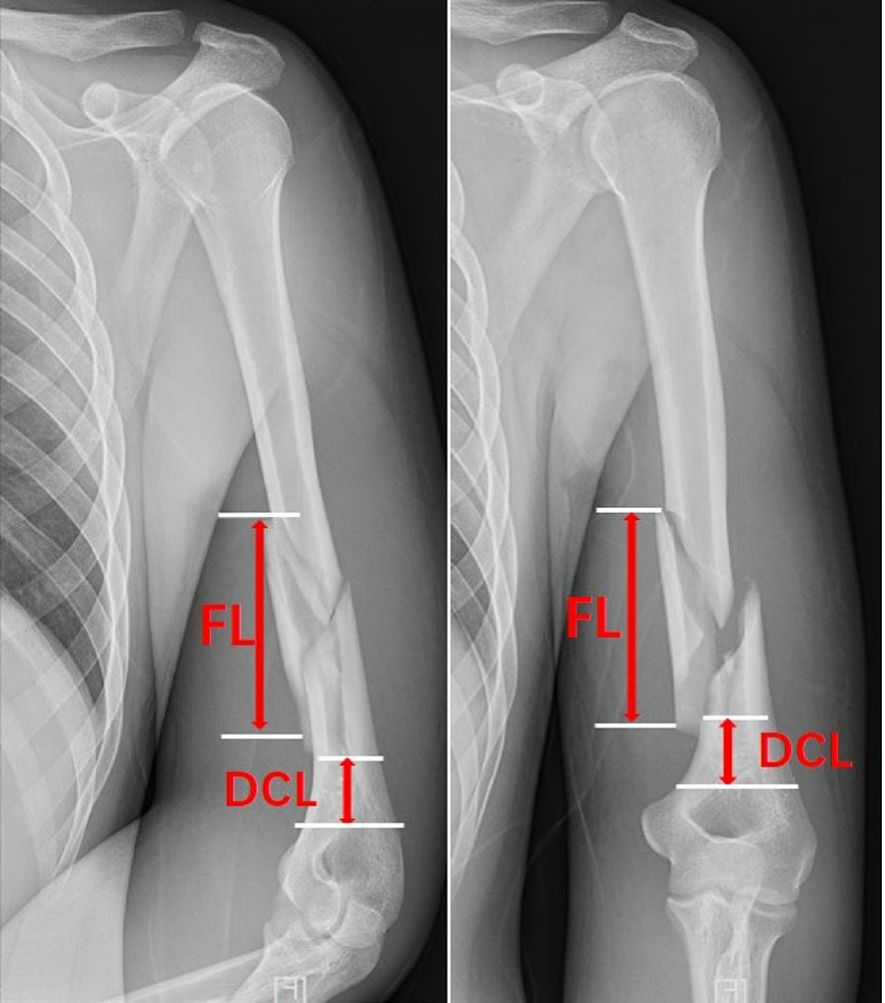
Positive and lateral X-ray of a typical distal third diaphyseal fracture of humerus: The mean fracture length (FL) is defined as the longest fracture line observed in the X-ray image; The distal cortical length (DCL) is defined as the distance between the upper edge of the olecranon and the distal end of the fracture line.

Thirty patients were seen between January 2017 and October 2022. Patients had a distal third diaphyseal fracture of the humerus and underwent open reduction and internal fixation with a double plate using the modified medial approach combined with MIPO techniques. Open fractures, pathological fractures, intercondylar fractures of the humerus, and fractures combined with radial nerve injuries were excluded. All patients had completed X-ray and 3D CT examinations before their operation. A 4.5 mm narrow locking compression plate (LCP; Synthes®, Swiss) and a 3.5 mm LCP reconstruction plate (Synthes®, Swiss) were used for fixation. The length of the plate was determined by the fracture line, ensuring that both plates were fixed with at least two screws at the distal fragments. The mean fracture length and distal cortical length were analyzed preoperation. The length of the fracture was defined as the longest fracture line observed in the X-ray image and the distal cortical length was defined as the distance between the upper edge of the olecranon and the distal end of the fracture line (Fig. 1).

### General information

There were 20 males and 10 females included. The average age was 34.4 ± 9.1 years (range 18–56 years). There were nine cases on the left and 21 cases on the right. Causes of injury: motor vehicle accident injury in eight cases, arm wrist injury in 10 cases, and fall injury in 12 cases. According to the AO/ASIF fracture classification, there were four A1 cases (13.3%), three A2 cases (10%), 12 B2 cases (40%), and 11 B3 cases (36.7%) (Table 1).

**Table 1:** Distal third diaphyseal fracture of humerus according to age, classification of fractures and fracture length outcomes.

Characteristics	Values (N = 20 patients)
Mean age (years ± SD)	34.4 ± 9.1
Sex	
Male, n (%)	20 (66.7%)
Female, n (%)	10 (33.3%)
Side	
Right humerus injury, n (%)	21 (70%)
Left humerus injury, n (%)	9 (30%)
Mean distal cortical length, n (mm)	38.3 ± 5.3
Mean fracture length, n(mm)	56.1 ± 7.2
Mean interval between injury to operation (days ± SD)	4.9 ± 1.0
Mean follow-up, n (months ± SD)	20.1 ± 3.8
Injury mechanism, n (%)	
Motor vehicle accident injury	8 (26.7%)
Arm wrist injury	10 (33.3%)
Falls from standing height	12 (40%)
AO classification, n (%)	
A1	4 (13.3%)
A2	3 (10%)
B2	12 (40%)
B3	11 (36.7%)

### Surgical technique

**Figure 2:**
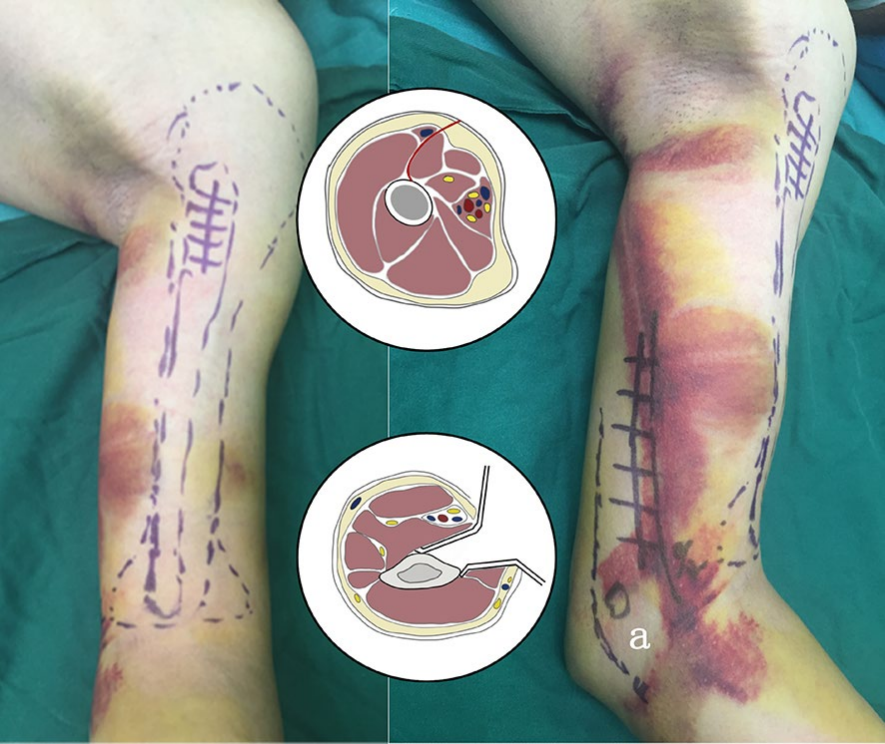
Operative incision and schematic diagram of the cross-sectional anatomy (a: Epicondyle of the humerus).

**Figure 3:**
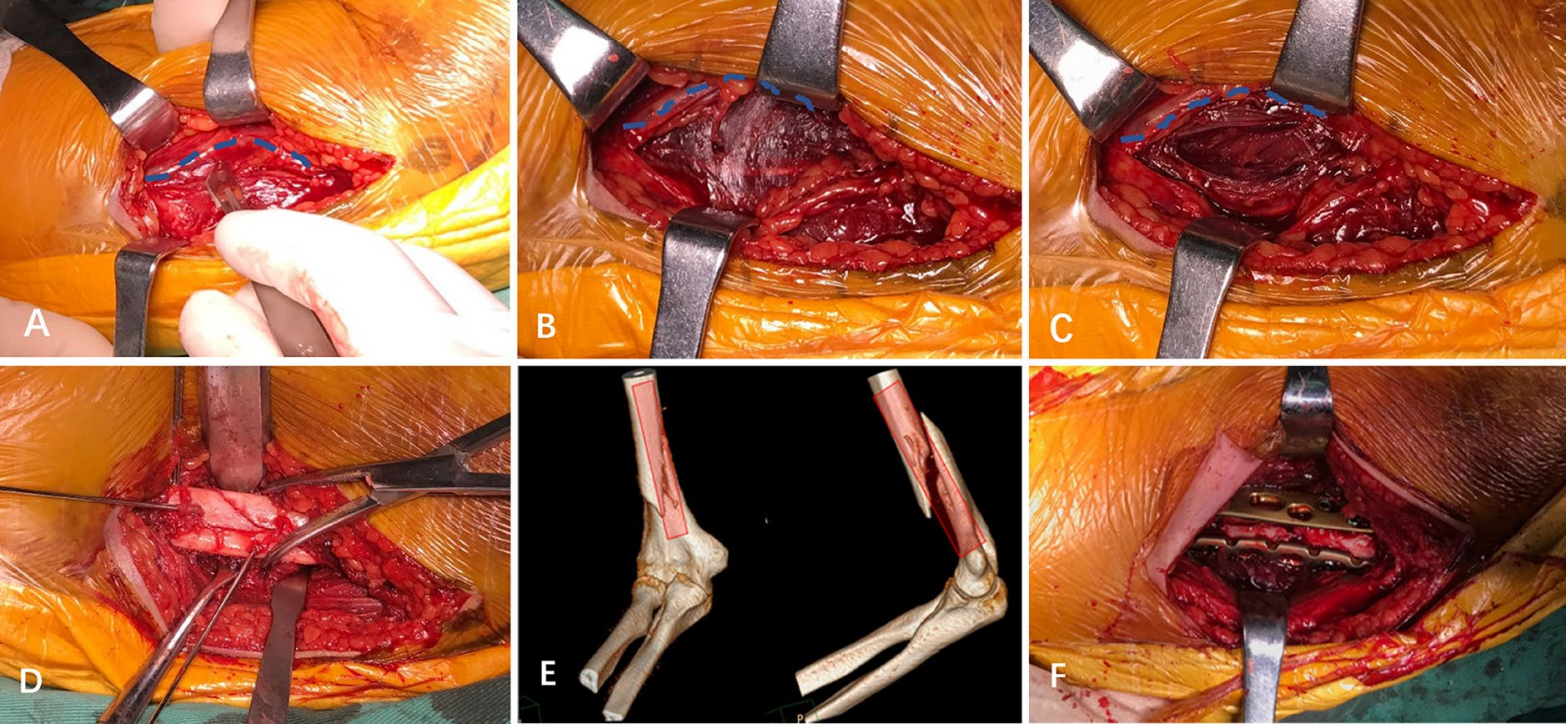
Exposure and fixation were performed by the modified medial approach. **(A)** The basilic vein was defined and exposed as a major marker (The blue dotted line is the basilic vein); **(B)** a gap between the biceps brachii and the medial part of the brachialis may be visible with careful blunted dissection of the bottom edge of the basilic vein; **(C)** the medial third side of the brachialis was incised in a longitudinal manner; **(D)** the fracture ends can be exposed, cleaned, reduction and temporarily fixed with kirschner wire. Visualization and subsequent release of radial never may be necessary if it becomes entrapped at the fracture site; **(E)** about 4/5 of the distal anterior area and the entire medial part of the distal humerus could be exposed (The red rectangular area in the figure is the exposure range); **(F)** anterior and the medial side plate were fixated perpendicular to the distal humerus.

**Figure 4:**
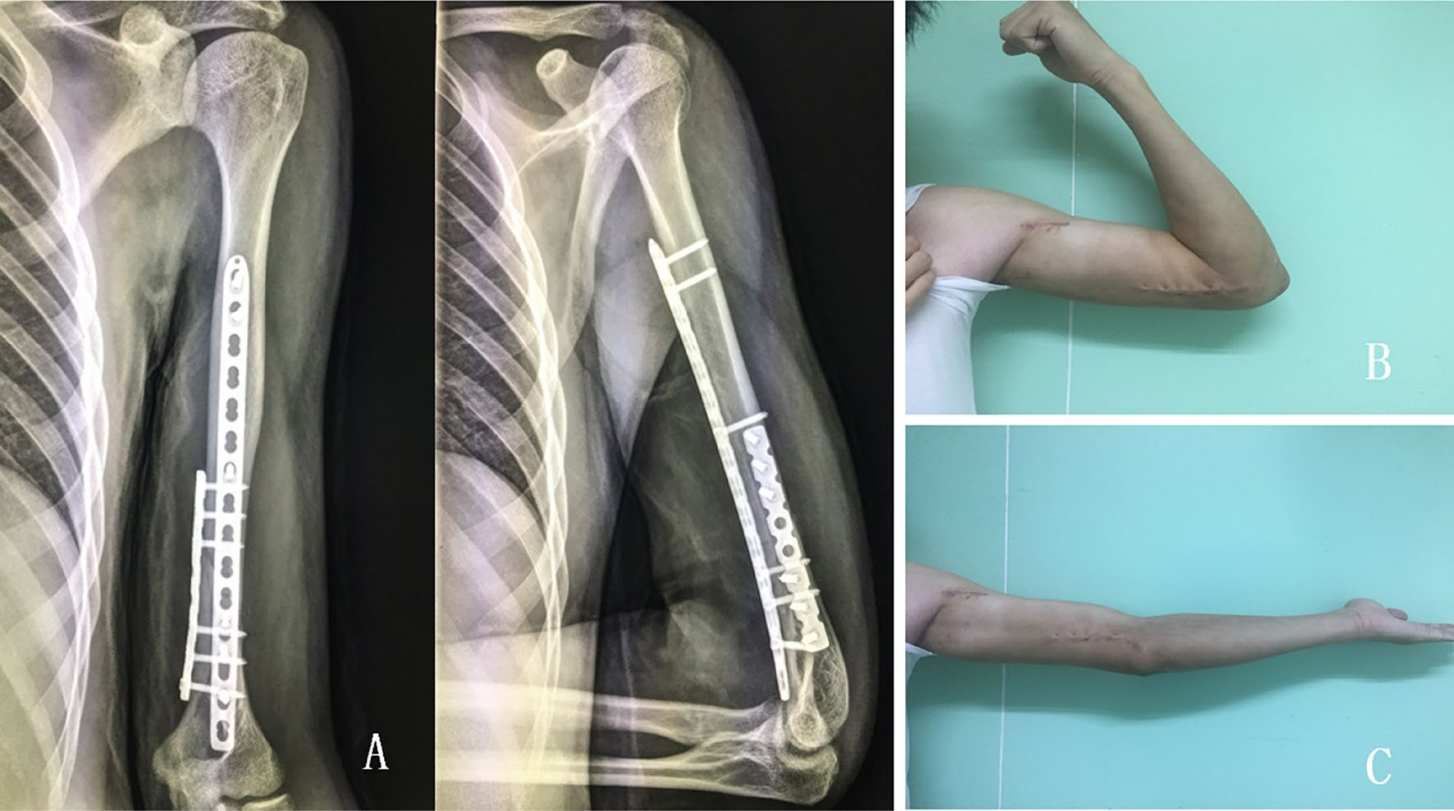
Clinical and radiological outcomes of a patient with distal third diaphyseal fracture of humerus. (**A**) The final follow-up X-ray; (**B,C**) the range of motion at the final follow-up of a 34 year-old male was shown.

The patient was placed in a supine position after general anesthesia with the upper arm placed at 90 degrees of abduction on a radiolucent table and the forearm in full supination (Fig. 2). For distal access, a longitudinal 5 cm incision was made at a position approximately 1.5 cm above the normal ulnar approach^11^. The basilic vein was first identified and protected (Fig. 3). A gap between the biceps brachii and the medial part of the brachialis may be visible with careful blunted dissection of the bottom edge of the basilic vein (Fig. 3). The medial third side of the brachialis was incised longitudinally, and the fracture ends were exposed, cleaned, reduced, and temporarily fixed with a Kirschner wire (Fig. 3). The ulnar nerve was indirectly protected by retracting the triceps brachii muscle posteriorly, while the brachialis muscle was pulled forward to protect other neurovascular structures. Approximately 4/5 of the distal anterior and the entire medial part of the distal humerus could be exposed using the modified medial approach. Visualization and subsequent release of radial never may be necessary if it becomes entrapped at the fracture site (Fig. 3). Internal fixation of the distal medial of the humerus was performed with a 3.5 mm LCP reconstruction plate, and two to three screws were used for proximal and distal fixation. Subsequently, the pectoralis major deltoid approach, which involves making an incision approximately 4 cm long between the proximal biceps brachialis muscle laterally and the deltoid muscle medially, was applied for the proximal incision. For the distal incision, a subbrachialis extraperiosteal tunnel was created by inserting a 4.5 mm narrow locking compression plate (LCP; Synthes®, Swiss), with a threaded drill sleeve attached as a handle, into the most distal hole for tunneling and plate insertion into the subbrachialis from the distal to the proximal incision. On the distal fracture part, two to three locking screws were inserted, and on the proximal fracture region, two to three screws were inserted (Fig. 4). Good fracture reduction, adequate plate placement, and right-length screws were all confirmed by intraoperative X-ray fluoroscopy. After extensive irrigation, the wound was closed in layers, and a rubber drainage was inserted. No external immobilization was used. Rehabilitation typically starts two days following surgery with passive forward flexion, aiming to achieve a goal of 0°–100° by the end of the second week to allow wound healing. Exercises for the pendulum function, raising, and shoulder joint abduction should all be performed in the same rehabilitation session. The elbow joint’s flexion and extension function were exercised with a partial load after six weeks. By three months, or when an X-ray image analysis revealed that the bones had healed, shoulder and elbow joint movement under heavy weightlifting was possible.

### Postoperative evaluation and management

We recorded the operation time, volume of bleeding, union time, and complications (whether there was a radial nerve, ulnar nerve, or musculocutaneous nerve injury). Standard anteroposterior and lateral views of the humerus were taken immediately following surgery and during the follow-up assessments at 4, 8, and 12 weeks postoperatively and at 6 and 12 months following surgery. The number of screws used and the fixed distal cortical point fragments were counted. The function of the shoulder was evaluated by Neer’s criteria^12^, which consisted of pain (none to totally disabled), function (strength, reach, and stability), range of motion (ROM) (flexion, abduction, extension, external rotation, and internal rotation), and anatomy (rotation, angulation, joint congruity, retracted tuberosities, metal failure, myositis, nonunion, and AVN). The function of the elbow was evaluated by Mayo’s criteria^13^, which is based on an assessment of pain (maximum score of 45 points), ulnohumeral motion (20 points), stability (10 points), and ability to perform five functional tasks (25 points).

### Ethics approval

All procedures in studies involving human participants were performed in accordance with the ethical standards of the institutional and/or national research committee and with the 1964 Helsinki Declaration and its later amendments or comparable ethical standards. Ethical approval was obtained from the Medical Ethics Review Committee of the Fuzhou Second Hospital, The Third Clinical Medical College, Fujian Medical University, Fuzhou Second Hospital of Xiamen University, School of Medicine, Xiamen University (approval No. 2023071).

### Consent to participate

Informed consent was obtained from all individual participants included in the study**.**

## Results

### Radiographic evaluation

The mean fracture length was 56.1 ± 7.2 mm and the distal cortical length was 38.3 ± 5.3 mm. Bone healing was observed by radiology in all patients by 10 to 16 weeks (average 12.1 ± 1.7 weeks) postoperatively, and there were no case of implant failure. The 7-hole 3.5 LCP reconstruction plate was the most commonly used, used in a total of 14 cases, and the 4.5 mm narrow LCP, including the 10-hole to 14-hole plates, was used in the other cases. In every case, the distal fragments were secured by a minimum of four screws and a maximum of 12 cortical points (average 8.9 ± 1.3). The mean number of screws used in the 4.5 mm LCP and 3.5 mm recon plate were 2.2 ± 0.4 and 2.3 ± 0.4 (Table 2).

**Table 2:** Plate length and mean number of screws, mean cortical points of screws fixed on distal fragment.

Parameters	Values
4.5-mm narrow LCP length (%)	
10 hole	9 (30%)
12 hole	13 (43.3%)
14 hole	8 (26.7%)
3.5-mm reconstruction LCP length (%)	
6 hole	8 (26.7%)
7 hole	14 (46.6%)
8 hole	8 (26.7%)
Mean number of screws fixed on distal fragments	
On 4.5-mm narrow LCP	2.2 ± 0.4
On 3.5-mm reconstruction LCP	2.3 ± 0.4
Mean cortical points of screws fixed on distal fragment	8.9 ± 1.3

### Clinical evaluation

All patients were operated on within 2–7 days (mean, 4.9 ± 1.0 days) of injury. The mean operative time was 84.8 ± 13.4 min (60–110 min). The mean blood loss during surgical treatment was 46.5 ± 10.2 ml (30–60 ml). The mean follow-up period was 20.1 ± 3.8 months (range 12–28 months). One patient exhibited poor surgical wound healing in the 5 days following the operation. Subsequent bacterial culture and relevant examinations were conducted to rule out infection, ultimately resulting in successful wound healing after debridement. There were no iatrogenic radial, ulnar, or musculocutaneous nerve injuries. At the final follow-up, all patients had good function of their shoulder and elbow. The maximum flexibility of the elbow was from 130° to 145° (average, 138.1 ± 4.8°), with a maximum flexibility straightness of 0° to 5° (average, 2.2 ± 1.3°). The Mayo elbow joint function score was 80–100 (average, 91.4 ± 5.0). The Neer shoulder joint function score was 85–100 (average, 92.5 ± 3.9) (Table 3) (Supplementary Fig. 1).

**Table 3:** Clinical and Functional outcomes of patients with distal third diaphyseal fracture of humerus.

Parameters	Values
Mean operative time ± SD (min)	84.8 ± 13.4
Mean blood loss ± SD (ml)	46.5 ± 10.2
Mean time to union ± SD (weeks)	12.1 ± 1.7
Complication (%)	1 (3.3%)
Elbow range of motion ± SD (°)	
Elbow extension	2.2 ± 1.3
Elbow flexion	138.1 ± 4.8
Mayo elbow joint function score ± SD	91.4 ± 5.0
Neer shoulder joint function score ± SD	92.5 ± 3.9

## Discussion

The surgical treatment of the distal third diaphyseal fracture of the humerus includes external fixation, intramedullary nail and plate fixation. Plate fixation has long been considered the most reliable treatment for such fractures^14^. However, it has been reported in the literature that the incidence of nonunion of these fractures is still as high as 5–15%^15^. Oliver et al.^16^ believe that 75% of treatment for humerus fractures in the distal third diaphyses failed due to the instability of the initial fixation. We believe that the instability of the initial fixation is primarily due to a lack of understanding of the difficulties regarding the treatment of these fractures. We reviewed this type of fracture and found that the farther down the fracture line, the more limited the internal fixation options. The average fracture length in the patients in this study was 56.1 ± 7.2 mm, and the average distal cortical length was 38.3 ± 5.3 mm. For such fractures, neither intramedullary fixation nor single-plate fixation could not provide an effective working distance and internal fixation stability. Therefore, we suggest specifically referring to this part of fractures as a distal third diaphyseal fracture of the humerus and that a stronger fixator be used for more stable initial fixation be to reduce the incidence of complications. Although conventional single-plate fixation has the advantage of less interference with soft tissue, blood vessels, and nerves^17,18^, it is often insufficient to fix the distal third diaphyseal fracture of the humerus with this fixation. Immobilization is often needed in the early postoperative period, thus losing the opportunity for early functional exercise. In many studies^19,20^ including biomechanical analysis, researchers have shown that a single plate is suitable for fixing fracture types of the middle and upper part of the humerus, while a double plate and double column fixation can provide better anti-bending and anti-torsion forces for fractures of the distal humerus. Since the fracture characteristics of the distal third diaphyseal fracture of the humerus determines if the distal end of the fracture is fixed with a single plate, it is difficult to achieve with three screws and six cortical fixations and the fixation can be unstable. In contrast, in double plate fixation, can ensure at least four screws and eight cortical fixation points beat the distal end of the fracture, thus providing strong resistance to torsional stress. Kosmopoulos et al.^21^ used biomechanical experiments to confirm this view. Early functional exercises of the elbow and shoulder are the key factors in achieving good postoperative function. Relevant studies have reported the use of a double plate in clinical practice and achieved satisfactory results, which also confirms this view^22,23^. The thirty patients in this article were treated with the modified medial approach combined with MIPO using a double-plate technique. At least four screws were fixed at the distal end of the fracture, and average (8–12) cortical fixation was achieved. Through strong fixation, the patient was able to start functional exercise of the shoulder and elbow joint on the second day after surgery. Because of the anatomical reduction and biomechanical stability, the fracture healed fast, in an average of 12.1 weeks. Early functional exercises helped to achieve good elbow flexion and extension function as well (Fig. 4).

Currently, surgical approaches for the treatment of distal third diaphyseal fractures of the humerus mainly include anterolateral, posterior, medial, direct anterior, and anterior MIPO approaches. Among them, the anterolateral approach is the most commonly used in clinical practice. However, this approach requires a large incision and routine exposure of the radial nerve, which often results in iatrogenic radial nerve injury^24^. Rafael-Kakazu et al.^25^ reported that the probabilities of iatrogenic radial nerve injury caused by the anterolateral approach, anterior approach, and posterior approach were 1/5, 1/25, and 1/9, respectively. The anterior MIPO technique approach has the advantage of avoiding the risk of iatrogenic radial nerve injury by not requiring exposure of the radial nerve. It also preserves the biological integrity of the fracture end, requiring a smaller incision, causing less trauma, limiting disruption of the blood supply, causing little intraoperative bleeding, achieving a high fracture healing rate, and ensuring good recovery of shoulder joint function^26^. However, it cannot be used for the anatomic reduction of fracture ends. In recent years, the ulnar approach to distal humerus fracture has gained popularity and is more commonly used in clinical practice^27^. However, due to the complexity of blood vessels and nerves in the medial incision, a high level of anatomical knowledge is needed to avoid causing iatrogenic injury, making this approach undesirable. Nevertheless, other researchers argue that the medial incision is not only concealed but also safe and minimally invasive^28^. Buranaphatthana reported the fixation of twenty cadaveric arms utilizing the anteromedial approach and 12-hole precontoured narrow locking compression plates (LCP) using the MIPO technique^11^. Between the biceps and deltoid muscles, a proximal approach was used to reach the bone. The brachialis was raised from the medial intermuscular septum during the distal approach. From distal to proximal, the plate was placed into the brachialis tube on the anteromedial face. In our study, we improved the medial approach through clinical studies. A longitudinal 5 cm incision was made at a position about 1.5 cm above the normal ulnar approach^11^. With the modified medial approach, approximately 4/5 of the distal anterior area and the entire medial part of the distal humerus were accessible. Internal fixation of the distal medial and anterior of the humerus can be performed with a 3.5 mm LCP reconstruction plate and a 4.5 mm Narrow locking compression plate. As a result, the distal humerus did not need to be supplemented with a skin incision, and if the anterior side plate is placed slightly to the side of the ulna, the distal screw may be inserted directly through the modified medial incision. While reducing the length of the surgical incision, it also avoided the risk of musculocutaneous nerve injury. As a result of strong fixation, we obtained better shoulder and elbow joint function.

The conventional anterior MIPO technique necessitates the division of the brachialis muscle, which may harm the radial nerve or muscular branches and denervate some muscle, resulting in motor weakness. The brachialis muscle was also separated in our investigation, but the aforementioned problems did not occur. We consider the following reasons our procedure avoided these complications: (1) The conventional anterior MIPO approach requires division of the brachialis muscle at both the distal and proximal ends, with the distal incision at the lateral radial. The brachialis muscle in these regions is where the branches of the radial nerve and musculocutaneous nerve pass through, so it is easily injured and leads to muscle paralysis^29^. However, we split only the medial 1/3 of the brachialis muscle, which is a relatively safe area. (2) Brachialis muscle scarring and inadequate postoperative rehabilitation may be involved in limiting the elbow range of motion with the conventional anterior MIPO approach. Our modified medial MIPO technique with a dual plate technique was able to allow early functional exercise and avoid complications.

The modified medial (MIPO) technique for the treatment of distal third diaphyseal fracture of the humerus has some limitations. First, this approach is not suitable for cases where the fragment is located on the radial side, and radial nerve injury needs to be explored. Second, the study had a limited sample size. Third, the potential for selection bias is due to the retrospective design of the study. Finally, this surgical approach has a long learning curve.

## Conclusion

Based on the above, our study shows that the modified medial approach combined with the anterior MIPO approach is advantages in that it is minimally invasive, allows strong fixation, and avoids iatrogenic radial nerve and ulnar nerve injury in the treatment of distal third diaphyseal fracture of the humerus. For such fractures, it is an optional treatment and worthy of clinical application.

### Supplementary Information

Supplementary Information.

## Data Availability

All data generated or analysed during this study are included in this published article and support our published claims and comply with field standards [Related files].
