# Supplementary material for: Modified medial minimally invasive double-plating osteosynthesis techniques for the treatment of distal third diaphyseal fracture of humerus
Source: Sci Rep. 2023 Dec 7;13:21621. doi: 10.1038/s41598-023-49111-3 (PMC10703802; doi:10.1038/s41598-023-49111-3)
Supplement: Supplementary file 1 — Supplementary Information. [file 41598_2023_49111_MOESM1_ESM.docx]

**Modified medial minimally invasive double-plating osteosynthesis techniques for the treatment of distal third diaphyseal fracture of humerus**

1. **Provides a good biomechanical stability and biological healing environment for fracture healing**

Although our surgical plan has two incisions and double plates are inserted for fixation, our proximal surgical incision is a minimally invasive incision to insert screws at the proximal end of the plate, which has no effect on the blood supply of the fracture end. Our modified medial incision eliminates the need for exposure of vital blood vessels and nerves during surgery, as they are effectively shielded by surrounding muscles, thus minimizing any potential injury. Our soft tissue injury was comparatively minimal when compared to certain single-incision surgical protocols that have been published (Fig. 1,2) [1]. Therefore, it provides a good biomechanical stability and biological healing environment for fracture healing.


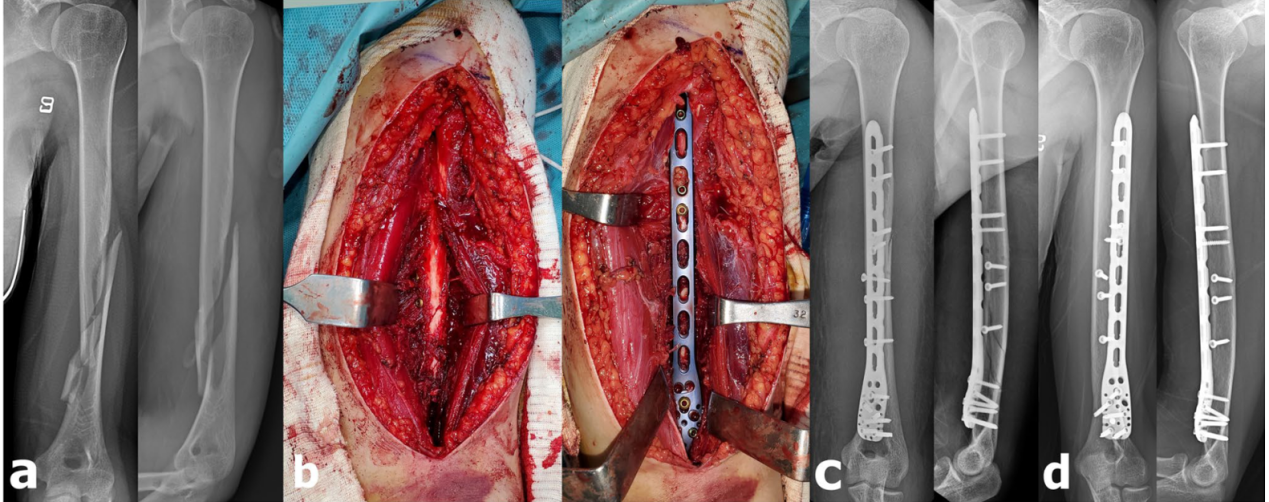


**Fig. 1** The anterior approach employed in treating distal third diaphyseal fracture of humerus necessitates extensive exposure, thereby posing a considerable risk of nerve injury and disruption to blood supply [1].


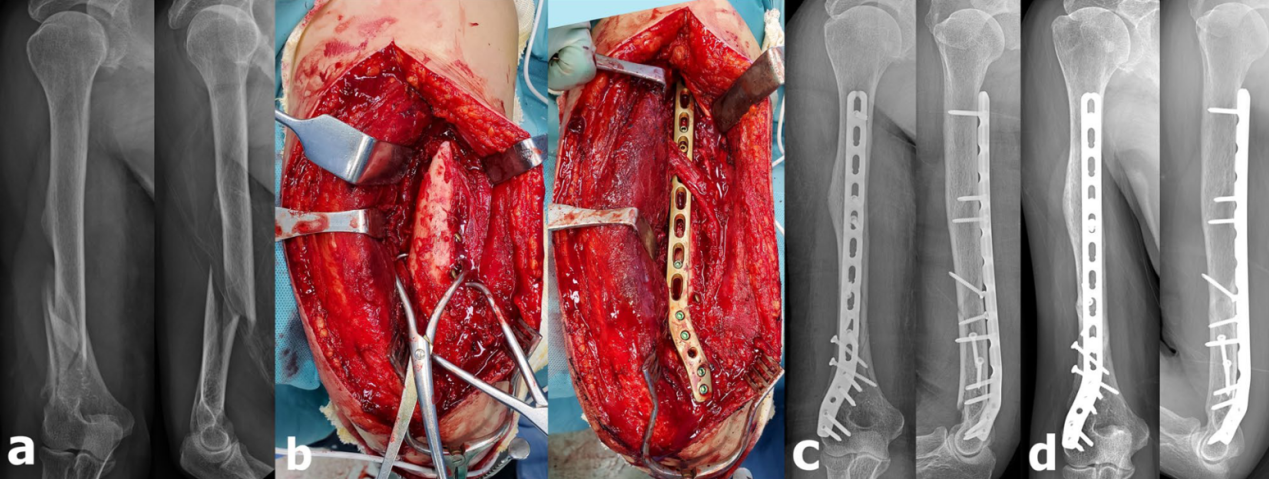


**Fig. 2** The posterior approach used to treat distal third diaphyseal fractures of the humerus requires extensive exposure, which in turn increases the risk of nerve injury and disruption to blood supply [1].

1. **The modified medial approach is the optimal incision solution for minimally invasive double plate fixation.**

MIPO approaches for distal third diaphyseal fractures of the humerus have been described using anterior [2–4], anterolateral [5–7], and posterior [8] approaches, each of which has both advantages and disadvantages. The anteromedial MIPO approach, Buranaphatthana[9] study it can be performed through the internervous plane beneath the brachialis muscle without exposing any nerves or vital blood vessels with a single 12-hole plate. However, this approach is limited in its ability to expose the anterior of the distal humerus, especially when it requires plate fixation. Therefore, a modified medial approach was proposed to provide a greater range of exposure in our study.​ We advocate dual plating for the treatment of such fractures. However, it is difficult to place a double plate for fixation in a single incision. If double plates are placed through a single incision, it requires a large surgical incision and a large soft tissue injury (Fig. 3) [2]. ​We performed direct exposure, reduction, and fixation of the fracture end via a modified medial surgical incision. With the modified medial approach, approximately 4/5 of the distal anterior area and the entire medial part of the distal humerus were accessible. ​As a result, the distal humerus did not need to be supplemented with a skin incision, and if the anterior side plate is placed slightly to the side of the ulna, the distal screw may be inserted directly through the modified medial incision. While reducing the surgical incision it also avoided the risk of musculocutaneous nerve injury. At the same time, we used the MIPO technique on the anterior side and placed a long plate to avoid damage to the posterior radial nerve by screws inserted from the anterior to the posterior side (Fig. 4). Our proximal incision is also minimally invasive with minimal soft tissue damage (Fig. 5). In the pictures we provide of the surgical manipulation, no vital blood vessels and nerves are involved throughout the procedure (Fig. 6). The biomechanical stability provided by the double plate, and the minimal invasive incision had minimal disruption to the blood supply of the fracture and provided a biological healing environment.


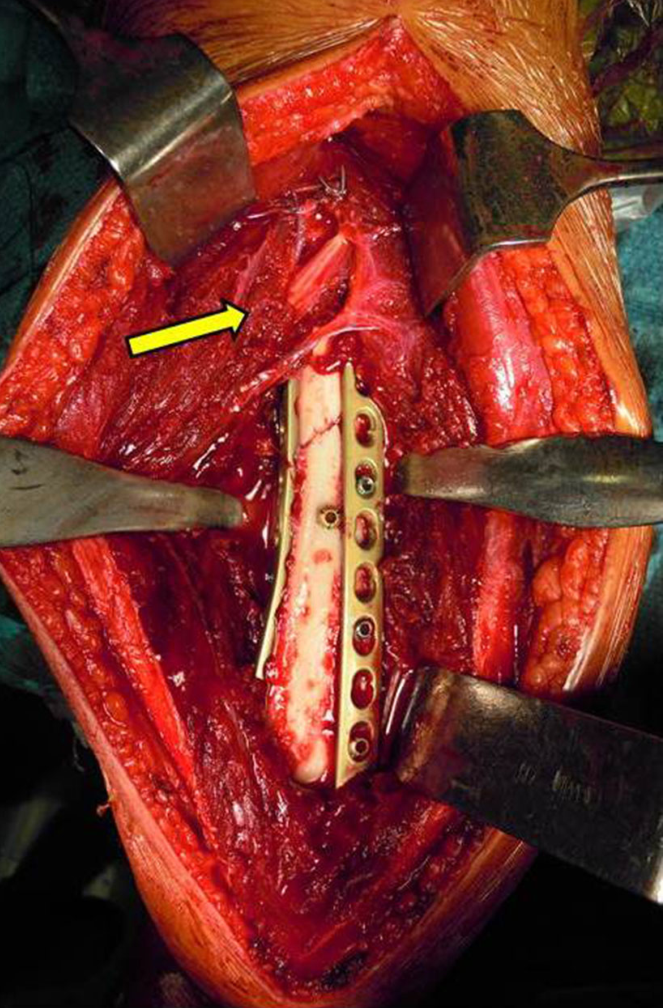


**Fig. 3** The posterior approach to split the triceps brachii muscle has large intraoperative trauma, and large muscle damage, and the radial nerve needs to be exposed. ​Such a large trauma will cause a disruption of the blood supply to the fracture site, which will affect the healing of the fracture site ^2^.


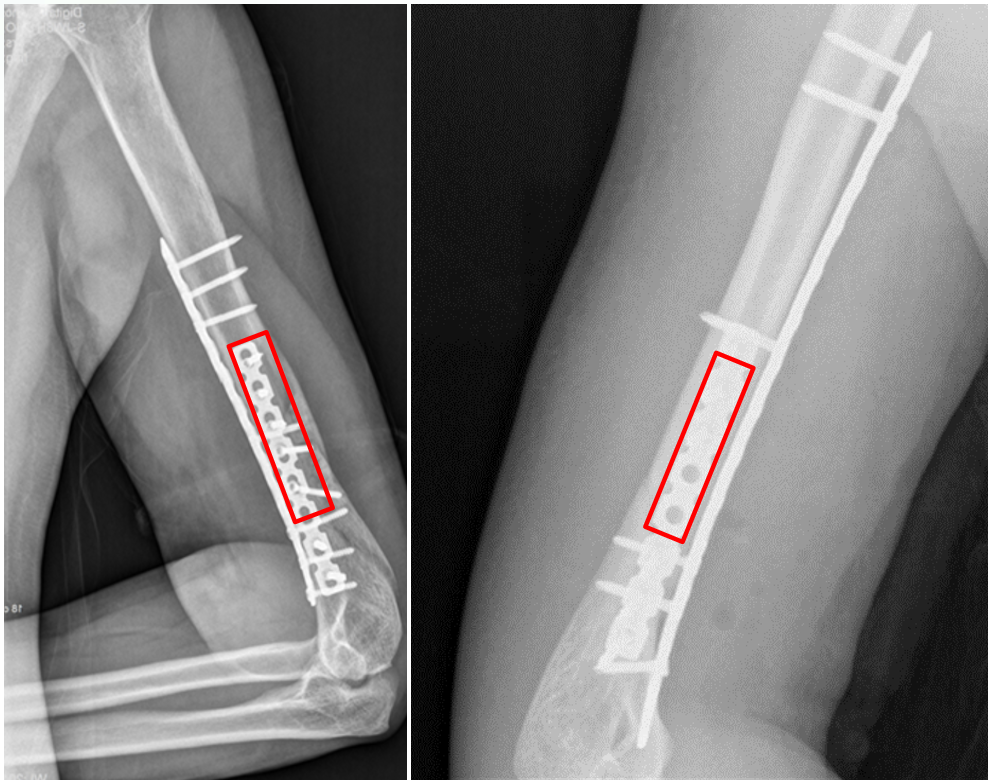


**Fig. 4** We used the MIPO technique on the anterior side and placed a long plate to avoid damage to the posterior radial nerve by screws inserted from the anterior to the posterior side.


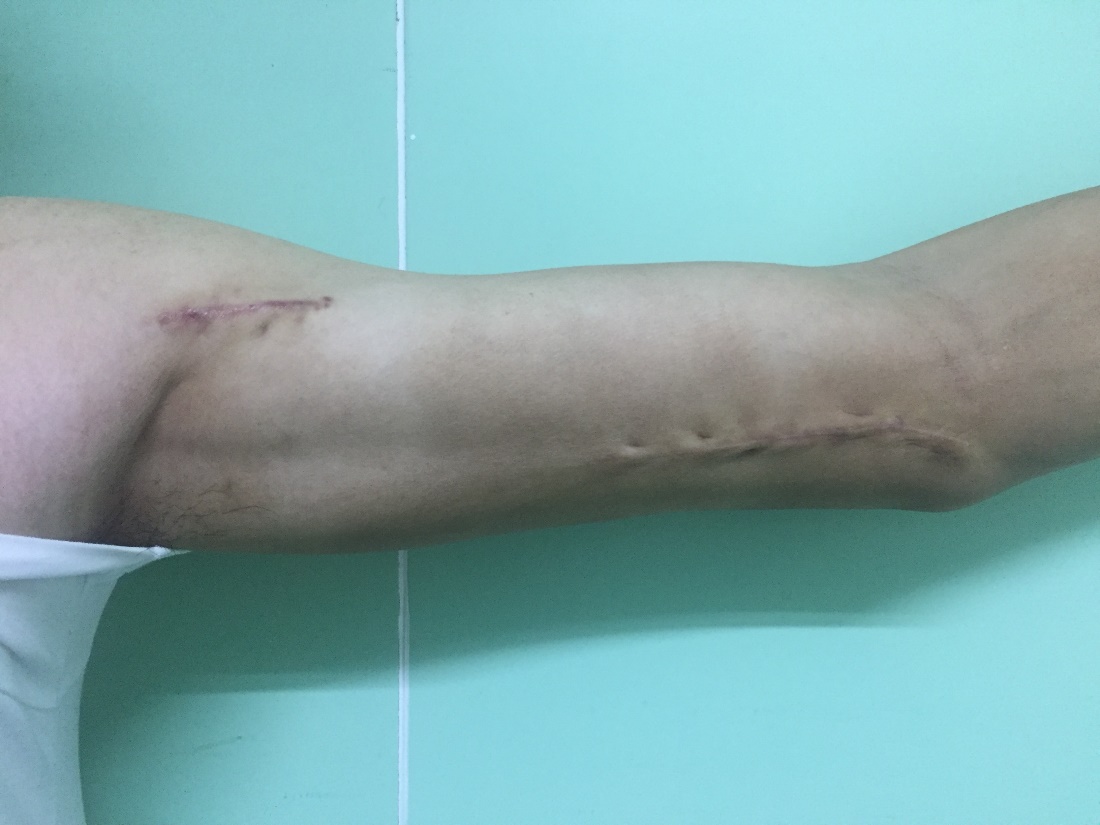


**Fig. 5** The proximal and distal incisions.


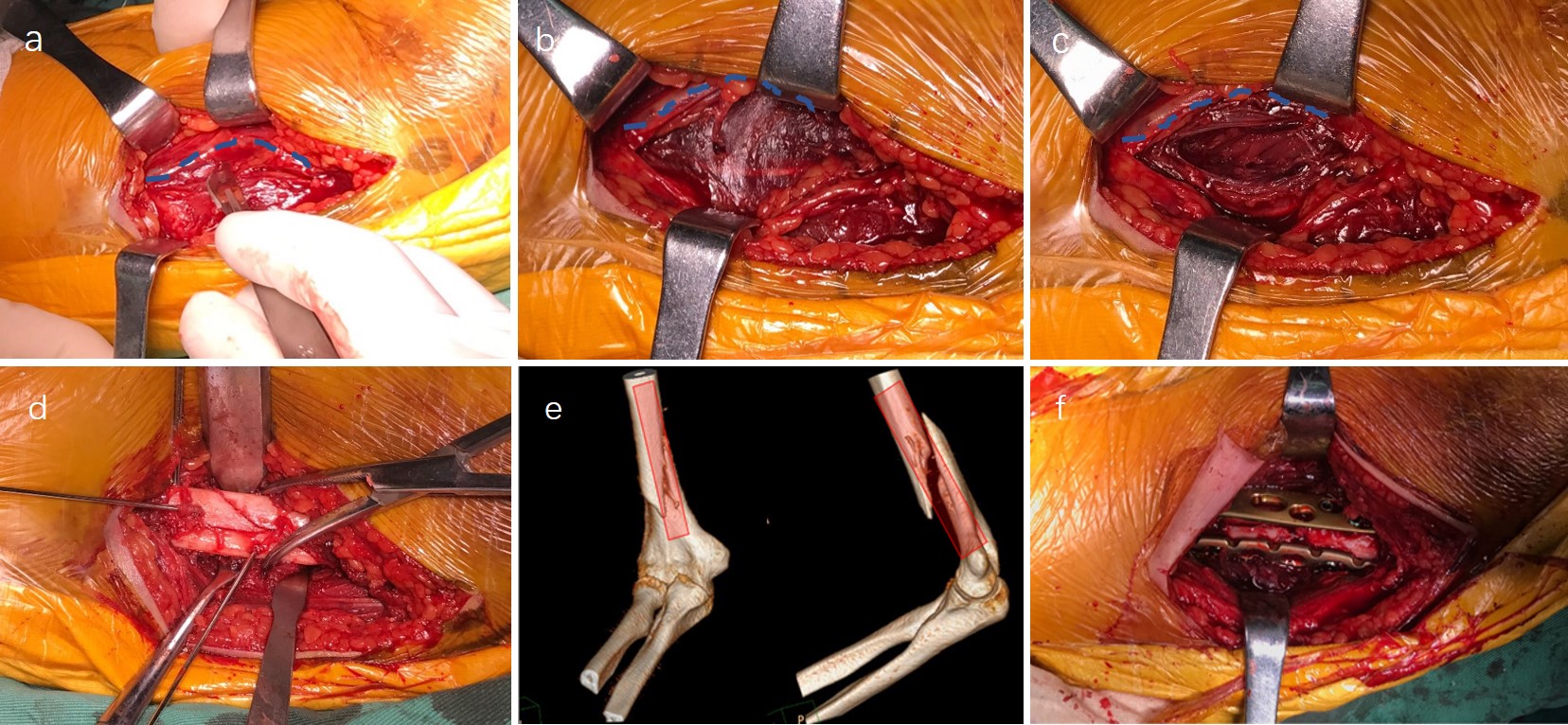
**Fig. 6**. Exposure and fixation were performed by the modified medial approach

1. **The Modified medial approach can minimize damage to blood vessels and nerves**

Although our surgical plan involves two incisions and the insertion of double plates for fixation, our proximal surgical incision is a minimally invasive approach. This incision, approximately 4cm in length, is positioned between the proximal biceps brachialis muscle laterally and the deltoid muscle medially. It allows for the insertion of screws at the proximal end of the plate without interfering with vital blood vessels and nerves. In contrast to the traditional ulnar approach or the lateral and posterior approaches, which require exposure of the radial nerve or ulnar nerve, our modified medial surgical approach does not require exposure of the radial nerve and ulnar nerve. We also performed a cadaver study before starting our clinical practice (Fig. 7). ​Moreover, we use long plates that span the radial nerve region and, in principle, minimize vascular nerve damage (Fig. 4). Because there is no need to expose the radial nerve and ulnar nerve, if the internal fixation is removed later, there is no disadvantage of soft tissue scar around the nerve. From another point of view, the probability of nerve injury during the second surgery is reduced. Our modified medial incision eliminates the need for exposure of vital blood vessels and nerves during surgery, as they are effectively shielded by surrounding muscles, thus minimizing any potential injury. On the contrary, it has been reported that the anterior MIPO approach has a high probability of intraoperative musculocutaneous nerve injury due to the splitting of the brachialis muscle on the radial side (Fig. 8) [11]. Therefore, we believe that this approach can minimize damage to blood vessels and nerves.


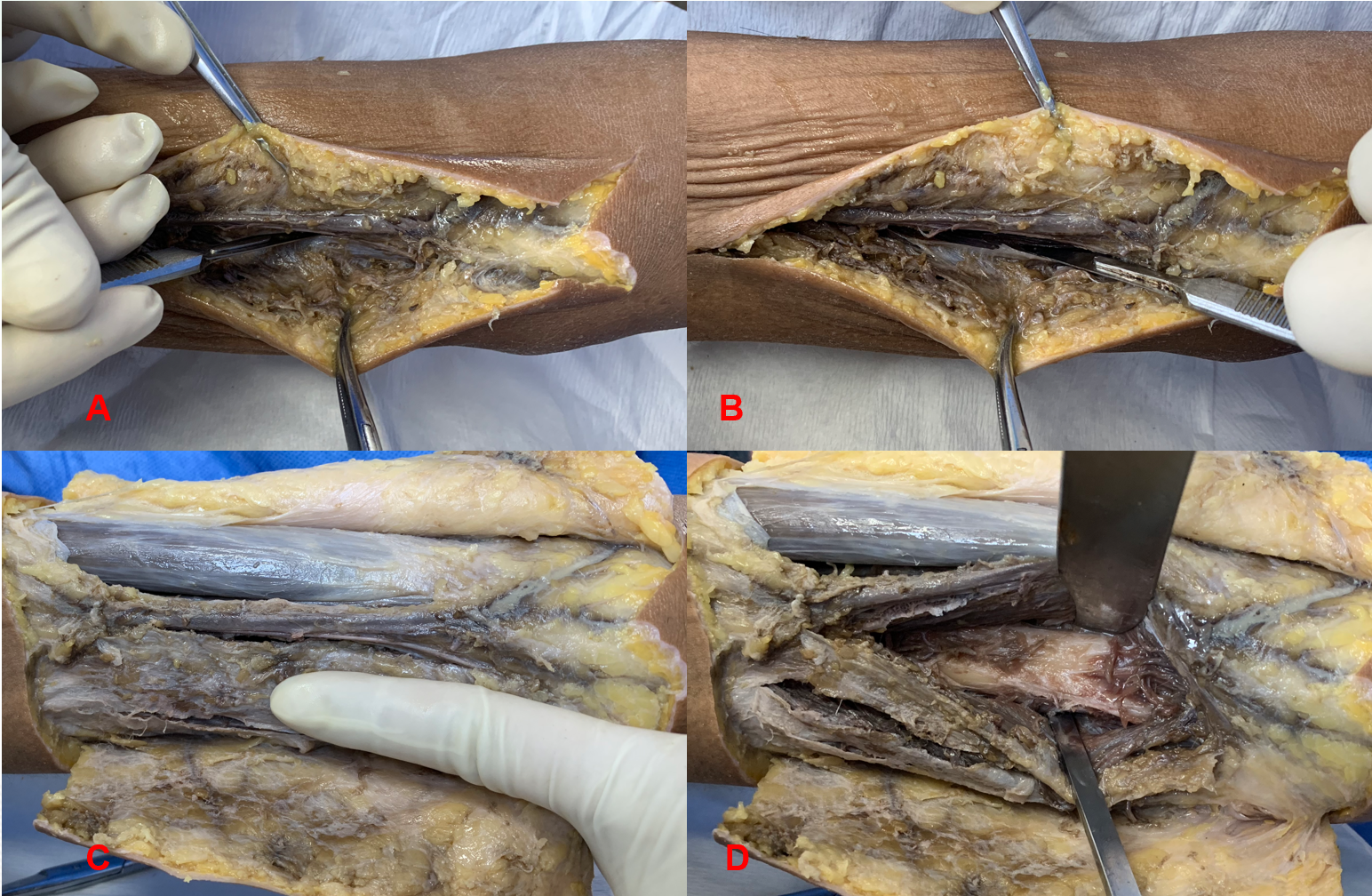


**Fig. 7** **(A)** The basilic vein was defined and exposed as major markers; **(B)** The medial third side of the brachialis was incised in a longitudinal manner; **(C)** ​The unexposed ulnar nerve is approximately one transverse finger away from the basilic vein; **(D)** ​The entire medial portion of the distal humerus and the distal anterior region can be exposed in cadaver studies.

**Fig. 8** The anterior Mipo approach has a high probability of intraoperative musculocutaneous nerve injury due to the splitting of the brachialis muscle on the radial side ^3^.

1. **Visualization and subsequent release of radial never may be necessary if it becomes entrapped at the fracture site**

**
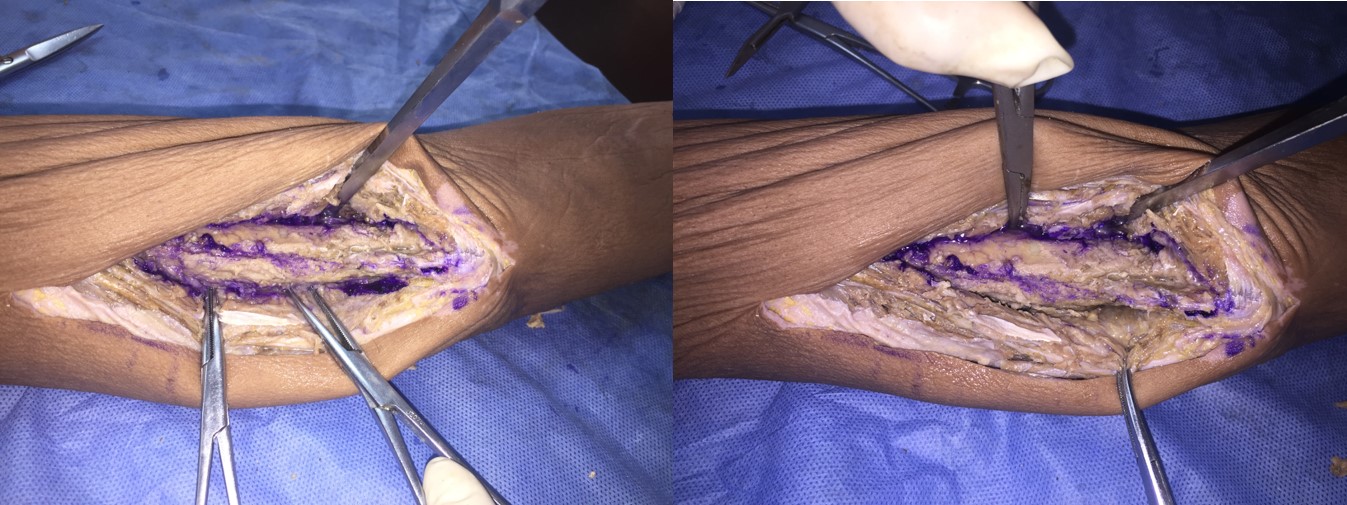
**In our Materials and Methods, we state that our case rules out radial nerve injury, which is equivalent to saying that radial nerve continuity is present in our included case. Of course, we cannot rule out cases in which the radial nerve is trapped at the site of the fracture, but there are no symptoms of radial nerve injury. However, our surgical approach can expose the entire fracture area and release the radial nerve if it is trapped in the fracture (Fig. 9,10).

**Fig. 9** About 4/5 of the distal anterior area and the entire medial part of the distal humerus could be exposed. If the radial nerve is trapped at the fracture site, it can also be visualized and subsequently released.

**
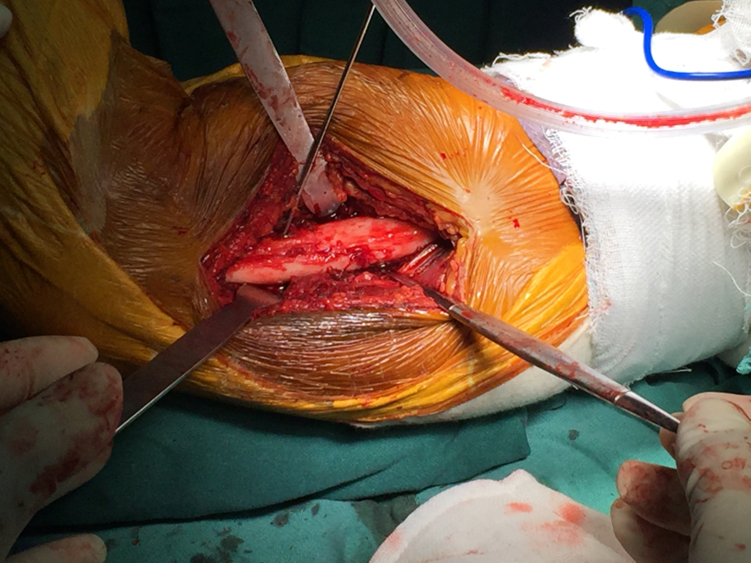
Fig. 10** About 4/5 of the distal anterior area and the entire medial part of the distal humerus could be exposed. If the radial nerve is trapped at the fracture site, it can also be visualized and subsequently released.

**R[eferences](javascript:;)**

1. Shin SJ, Kwak JW, Sohn HS. Comparison between anterior and posterior plating systems in extra-articular distal-third diaphyseal humeral fractures. Int Orthop. **46**(9):2119-2126 (2022).
2. Apivatthakakul T, Arpornchayanon O, Bavornratanavech S. Minimally invasive plate osteosynthesis (MIPO) of the humeral shaft fracture. Is it possible? A cadaveric study and preliminary report. Injury. **36**(4):530–8 (2005).
3. Zhiquan A, Bingfang Z, Yeming W, Chi Z, Peiyan H. Minimally invasive plating osteosynthesis (MIPO) of middle and distal third humeral shaft fractures. J Orthop Trauma. **21**(9):628–33 (2007).
4. Kobayashi M, Watanabe Y, Matsushita T. Early full range of shoulder and elbow motion is possible after minimally invasive plate osteosynthesis for humeral shaft fractures. J Orthop Trauma. **24**(4):212–6 (2010).
5. Zogbi DR, Terrivel AM, Mouraria GG, Mongon ML, Kikuta FK, Filho AZ. Fracture of distal humerus: MIPO technique with visualization of the radial nerve. Acta Ortop Bras. **22**(6):300–3 (2014).
6. Lee T, Yoon J. Newly designed minimally invasive plating of a humerus shaft fracture; A different introduction of the plate. Int Orthop. **40**(12):2597–602 (2016).
7. Gallucci GL, Boretto JG, Alfie VA, Donndorff A, De Carli P. Posterior minimally invasive plate osteosynthesis (MIPO) of distal third humeral shaft fractures with segmental isolation of the radial nerve. Chirurgie de la Main. **34** (5):221–6 (2015).
8. Jiamton C, Ratreprasatsuk N, Jarayabhand R, Kritsaneephaiboon A, Apivatthakakul T. The safety and feasibility of minimal invasive plate osteosynthesis (MIPO) of the posterior aspect of the humerus: a cadaveric study. Clin Anatomy. **32**(2):176-182 (2019).
9. Buranaphatthana T, Apivatthakakul T, Apivatthakakul V. Anteromedial minimally invasive plate osteosynthesis (MIPO) for distal third humeral shaft fractures -Is it possible? : A cadaveric study. Injury. **50**(6):1166-1174 (2019).
10. Maresca A, Fantasia R, Cianforlini M, Giampaolini N, Cerbasi S, Pascarella R. Distal-third diaphyseal fractures of the humerus:choice of approach and surgical treatment. Musculoskelet Surg. **100**(Suppl 1):97-104 (2016).
11. Apivatthakakul T, Patiyasikan S, Luevitoonvechkit S. Danger zone for locking screw placement in minimally invasive plate osteosynthesis (MIPO) of humeral shaft fractures: A cadavefic study. Injury. **41**(2): 169-172 (2010)．
